# Mutant and curli-producing *E. coli* enhance the disease phenotype in a hSOD1-G93A mouse model of ALS

**DOI:** 10.1038/s41598-023-32594-5

**Published:** 2023-04-12

**Authors:** Zimple Kurlawala, Joseph D. McMillan, Richa A. Singhal, Johnny Morehouse, Darlene A. Burke, Sophia M. Sears, Eleonora Duregon, Levi J. Beverly, Leah J. Siskind, Robert P. Friedland

**Affiliations:** 1 Department of Neurology, University of Louisville, Louisville, KY 40202 USA; 2 Department of Neuroscience, University of South Florida, Tampa, USA; 3 KY IDeA Networks of Biomedical Research Excellence Bioinformatics Core, University of Louisville, Louisville, KY 40202 USA; 4 Kentucky Spinal Cord Injury Research Center, University of Louisville, Louisville, KY 40202 USA; 5 Department of Pharmacology and Toxicology, University of Louisville, Louisville, KY 40202 USA; 6 National Institute on Aging, Translational Gerontology, NIH, Bethesda, USA Maryland; 7 School of Medicine, University of Louisville, Louisville, KY 40202 USA

**Keywords:** Microbiome, Amyotrophic lateral sclerosis

## Abstract

The gut microbiome is a potential non-genetic contributing factor for Amyotrophic Lateral Sclerosis. Differences in gut microbial communities have been detected between ALS subjects and healthy controls, including an increase in *Escherichia coli* in ALS subjects. *E. coli* and other gram-negative bacteria produce curli proteins, which are functional bacterial amyloids. We examined whether long-term curli overexposure in the gut can exacerbate the development and progression of ALS. We utilized the slow-developing hSOD1-G93A mouse model of ALS with their C57BL/6J WT littermate controls, including males and females, with a total of 91 animals. These mice were on a normal chow diet and fed curli-producing or curli-nonproducing (mutant) *E. coli* in applesauce (vehicle) 3 times/week, from 1 through 7 months of age. Male hSOD1 mice demonstrated gradual slowing in running speed month 4 onwards, while females exhibited no signs of locomotive impairment even at 7 months of age. Around the same time, male hSOD1 mice showed a gradual increase in frequency of peripheral CD19^+^ B cells. Among the male hSOD1 group, chronic gut exposure to curli-producing *E. coli* led to significant shifts in α- and β-diversities. Curli-exposed males showed suppression of immune responses in circulation, but an increase in markers of inflammation, autophagy and protein turnover in skeletal muscle. Some of these markers were also changed in mutant *E. coli*-exposed mice, including astrogliosis in the brainstem and demyelination in the lumbar spinal cord. Overall, chronic overexposure to a commensal bacteria like *E. coli* led to distant organ pathology in our model, without the presence of a leaky gut at 6 months. Mechanisms underlying gut-distant organ communication are of tremendous interest to all disciplines.

## Introduction

Amyotrophic lateral sclerosis (ALS) is a rapidly progressive, fatal neuromuscular degenerative disease, with symptoms developing between the ages of 40–70 years. Current treatments modestly slow the progression of the disease by inhibiting glutamate release (Riluzole) and inhibiting neuronal death (Relyvrio) but there is no known cure. ALS is familial in only ~ 10% of cases, and can affect anyone worldwide regardless of racial, ethnic, or socioeconomic status. In the majority of cases, the initiating factor responsible for the illness is not known. The heterogeneity of possible risk factors such as intense exertion, toxins, metals, chemicals, trauma, electromagnetic field exposure, and military service point to the involvement of environmental factors in the etiology of ALS^1^.

The human gut microbiota is a rich source of metabolites, essential nutrients, and sometimes pathogens and play a role in digestion, regulation of the immune system and drug activation. In this way, the microbes inhabiting our body are a potent and long-lasting environmental influence that affect health. The gut-brain axis is of significant interest to the neurodevelopmental and neurodegeneration field and is actively being investigated in Parkinson’s disease^2–10^, Alzheimer’s^11–14^, ALS^15–17^, and autism spectrum disorders^18,19^. The imbalance in gut microbial communities is referred to as dysbiosis and can occur due to changes in diet and lifestyle^20^. Small intestinal bacterial overgrowth, one form of dysbiosis, is often found in older adults and can manifest with diarrhea, malabsorption, nutritional deficiencies, osteoporosis, and weight loss^21^. Most of the time, bacterial overgrowth does not cause discernible symptoms but may contribute to chronic pathologies such as cancer^22–24^. Recent work has suggested an association of abnormal gut microbial communities and ALS^15–17,25–27^. In germ-free G93A mouse models of ALS, Blacher et al. reported that supplementation of *Ruminococcis torques* and *Parabacteroides distasonis* worsened the ALS phenotype while *Akkermansia muciniphila* improved it^15^. In a similar mouse model of ALS, Wu et al. demonstrated a leaky gut and microbial dysbiosis which were restored by supplementation of butyrate, a short-chain fatty acid^26^. Clinical data from 50 ALS patients and 50 healthy controls showed a significant increase in the gut *E. coli* population in ALS patients^16,17^.

*E. coli* and other gram-negative bacteria produce curli protein fibrils. Bacterial curli consists of the major subunit CsgA which form cross-beta sheets aiding bacteria to form biofilms and enhance adhesion to surfaces. CsgA shares structural similarity to aggregation-prone amyloidogenic proteins like amyloid-β and α-synuclein. We and others have shown that gut exposure to curli, enhances α-synuclein misfolding in neurons of *C. elegans* and rodent models of Parkinson’s disease^28,29^. Most recently, Wang et al. have provided evidence that curli proteins can be uptaken from culture media by human neuroblastoma cell lines and gut-obtained curli can colocalize with α-synuclein in distant neurons^27^. Additionally, they reported that curli proteins are capable of cross-seeding α-synuclein, β-amyloid, huntingtin proteins as well as the ALS implicated SOD1-G85R protein in *C. elegans*^27^. Role of* E. coli* and curli fibers has also been reported in auto-immune diseases like Type 1 Diabetes^30^. 

In this study, we investigated whether overexposure to curli would exacerbate features of ALS in mice. We utilized the slow-developing hSOD1-G93A mouse model of ALS and exposed them to curli producing *E. coli*, mutant control (*E. coli* lacking curli) and vehicle for 6 months. We examined locomotive alterations every month using TreadScan equipment, markers of skeletal muscle atrophy (autophagy, E3 ligases, inflammation, skeletal muscle weights), neuroinflammation (cholinergic neurons, astrogliosis, microgliosis and demyelination), peripheral blood immunophenotyping (innate and adaptive immune cells), functional assessments of gut permeability, gastric motility, and fecal metagenomic analyses.

This manuscript provides evidence that chronic feeding of *E. coli* (both, curli-producing and mutant) in the presence of mice’s native gut microbiota produced bacterial dysbiosis and exacerbated markers of skeletal muscle atrophy in an hSOD1-G93A transgenic mouse model of ALS. Investigating underlying mechanisms did not fall within the scope of this manuscript and will be researched for future studies.

## Results

### Study design

**Figure 1:**
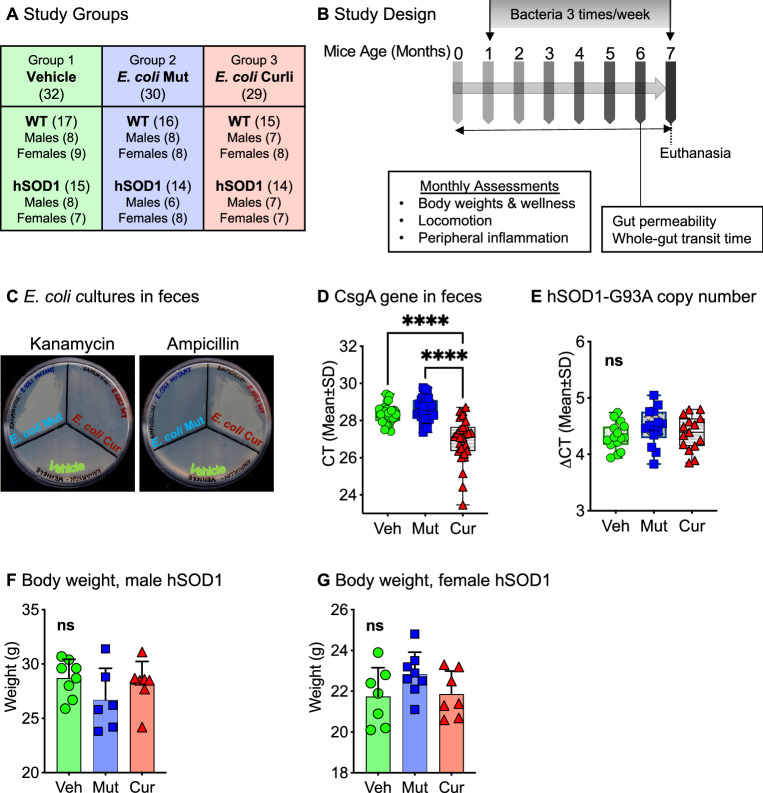
Study design. (**A**) There were a total of 91 animals in 3 main experimental groups—Group 1: Vehicle (applesauce only), Group 2: *E. coli* mutant in applesauce (lacking CsgA gene), Group 3: *E. coli* curli in applesauce (CsgA/curli producing). (**B**) Mice were assessed at baseline (3 weeks of age) and monthly for several functional measures as indicated. Feeding of bacteria or vehicle was started at 1 month of age, three times/week, for 6 months. (**C**) Confirmation of feeding—bacteria from fecal pellets were cultured in kanamycin and ampicillin agar plates, demonstrating resistance of *E. coli*-mutant to kanamycin and *E. coli*-curli to ampicillin antibiotics, respectively. (**D**) Confirmation of feeding—presence of CsgA gene were evaluated in DNA isolated from feces. qPCR was run from equal starting amount of DNA from all 3 feeding groups, which detected overexpression of CsgA in the curli-fed group, as well as endogenous expression in the vehicle and mutant groups (**E**) Copy numbers of hSOD1-G93A transgene in mice in all 3 feeding groups were not significantly different (**F** and **G**) There were no significant differences in body weights of mice at 7 months of age between bacterial fed groups. ns = not significant, ****p < 0.001, one-way ANOVA, n = 6–8 per group.

We utilized the modified hSOD1-G93A mouse model of familial ALS on a C57BL/6 J background^31^. This hSOD1-G93A strain exhibits an ALS motor phenotype at 6–7 months of age as it has a reduced copy number of the transgene, compared to the original strain which develops the ALS phenotype at 3 months (see “Methods” Section). We analyzed a total of 91 animals (Fig. 1A). These mice were on a normal chow diet and were fed 10^10^ CFU of curli-producing or curli non-producing (mutant) *E. coli* in applesauce (vehicle) 3 times/week from 1 through 7 months of age (see “Methods” Section, also previously described^29^). Overall, three groups were assessed: (1) vehicle only (applesauce only, no bacteria added); (2) mutant *E. coli* (lacking the CsgA operon, *E. coli* K-12 BW25113), and (3) wild-type *E. coli* (BW25113^32^ bearing the CsgA operon, involved in the production of curli), referred to as *E. coli* curli in this manuscript. Measures of wellness, locomotion and peripheral inflammation were evaluated every month (Fig. 1B). There were no significant differences in applesauce consumption between genotype (Supp. Fig. 1A,B), sex (Supp. Fig. 1C,D) or feeding groups (Supp. Fig. 1E–H). *Escherichia coli* were accurately detected in fecal pellets of mice that were fed bacteria with respective antibiotic resistance—mutant *E. coli* on Kanamycin resistance and curli-producing *E. coli* on Ampicillin resistance agar plates (Fig. 1C). Additionally, cycle threshold (CT) values for CsgA gene by qPCR confirmed overexpression of the gene in feces of the *E. coli* curli group (Fig. 1D). There were no differences in copy number of the hSOD1-G93A transgene for any bacterial feeding groups (Fig. 1E) or sex of mice (Supp. Fig. 1I). For all assessments in this study, we analyzed data for males and females separately as they develop the ALS phenotype at different rates, with males showing signs of motor impairment sooner than females^33^, also demonstrated in our study (Fig. 3A–D). There were no significant effects of bacterial feeding on body weights of mice at 7 months of age (Fig. 1F,G).

### Chronic gut exposure to curli-producing *E. coli* led to significant shifts in alpha and beta diversities of bacteria and viruses

**Figure 2:**
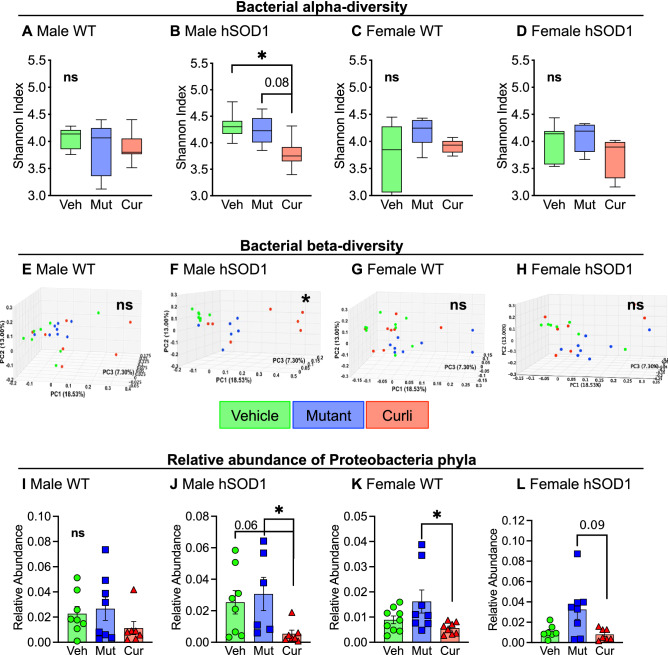
Whole-genome shallow shotgun sequencing analyses of fecal pellets at 6 months. (**A**–**D**) Within-group bacterial species diversity measured with Shannon alpha-diversity index demonstrated poorer species richness and abundance in curli-fed animals in the male hSOD1 cohort, *p < 0.05, Wilcoxon Rank Sum test. (**E**–**H**) Between-group bacterial beta-diversity measured with JACCARD index demonstrated distinct clustering of curli-fed animals in the male hSOD1 group. **p = 0.001, PERMANOVA test. (**I**–**L**) Relative abundance of *Proteobacteria* phyla were significantly reduced in curli-fed male hSOD1 and female WT groups, *p < 0.05, Kruskal–Wallis test. n = 6–8 per group.

Fecal pellets collected from mice at 6 months of age were submitted for whole-genome shallow shotgun sequencing. We assessed the influence of the three feeding groups on microbial diversity, composition, and taxonomic alterations. Diversity within individual samples also known as alpha-diversity was evaluated using Shannon index (measure of species abundance and evenness). We analyzed all 4 groups individually, namely male WT (Fig. 2A,E), male hSOD1 Fig. 2B,F), female WT (Fig. 2C,G) and female hSOD1 (Fig. 2D,H). Significant differences in alpha and beta diversities were detected only within the male hSOD1 group. Bacterial diversity was significantly decreased in the curli group compared to both vehicle (*p* < 0.05) and mutant (trending, *p* = 0.08) groups (Fig. 2B). Compositional changes between the samples were evaluated by the beta-diversity measure, JACCARD index, a similarity distance matrix. For bacterial species, the male hSOD1 cohort showed significant clustering of curli-fed animals (Fig. 2F), *p* = 0.001 calculated by PERMANOVA analyses. Alterations in taxonomy were assessed by comparing quantitative relative abundance between groups (Fig. 2I–L). In the male hSOD1 group (Fig. 2J), relative abundance of phylum *Proteobacteria* were significantly decreased in *E. coli* curli group compared to mutant and trending (*p* = 0.06) compared to vehicle group.

Alpha and beta-diversities for viruses were also assessed (Supp. Fig. 2A–H). At the level of viral phylogeny, male hSOD1 mice showed significant expansion of gammaretrovirus genera (Supp. Fig. 2I) compared to male WT animals. This difference was not observed in females (Supp. Fig. 2J). Depletion and enhancement of several bacterial (Supp. Fig. 3A) and viral strains (Supp. Fig. 3B) were observed in response to bacterial feeding, and a detailed list of significantly altered strains is provided in supplementary data.

Overall, these results indicate that after a 6-month gut exposure to *E. coli*, (i) bacterial alpha diversity was significantly decreased by *E. coli* curli feeding in the male hSOD1 cohort, (ii) microbial composition is perturbed by *E. coli* curli feeding in the male hSOD1 cohort and (iii) chronic expression of *E. coli* in the gut led to decreased relative abundance of phylum *Proteobacteria.*

### Curli-fed hSOD1 males showed locomotive abnormalities measured with TreadScan

**Figure 3:**
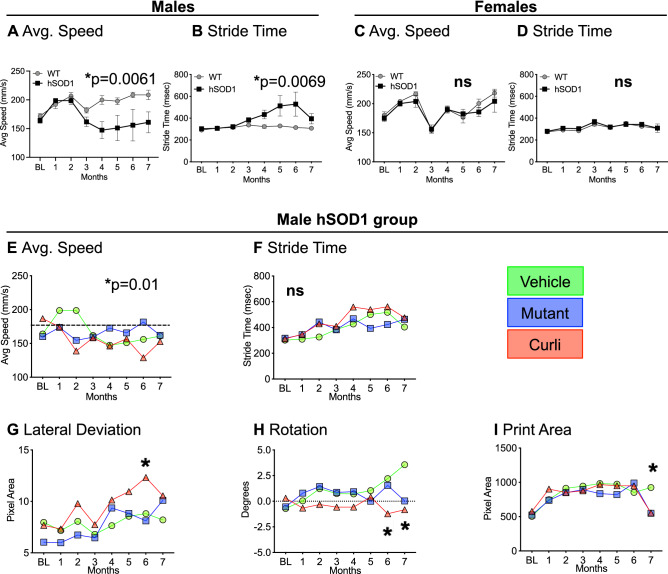
Gait kinematics and locomotion characteristics. (**A**–**D**) Average running speed and stride time measured with TreadScan analyses showed significant slowing of pace in hSOD1 males compared to WT controls (**A**). This finding was absent in females (**C**). Stride time, defined as the time elapsed between two successive initiations of stances, showed significant increases in hSOD1 males compared to WT controls (**B**). This finding was absent in females (**D**). Data represented as mean + /-SEM for (**A**–**D**). (**E**) Within the male hSOD1 cohort, the ANOVA was significant for effect of time on running speed, but bacterial feeding did not have a significant effect. (**F**) Bacterial feeding also did not significantly effect stride time. Within the male hSOD1 group**,** curli-fed mice showed significant differences for maximum lateral deviation of hind feet from the axis of the body (**G**), inability to efficiently move body from a straight axis (**H**), and a smaller print area of the hind feet (**I**) towards later months. *p < 0.05, Repeated measures two-way ANOVA or mixed-effects model analyses (REML) with Tukey’s multiple comparisons. n = 6–8 per group.

Starting at 3 weeks of age up to 7 months, we measured gait kinematics every month utilizing a treadmill-based tool called TreadScan^34^. Gait characteristics measured with TreadScan assessed gradual development of locomotor abnormalities. There was significant slowing of running speed in hSOD1 male mice starting at 4 months of age compared to WT males (Fig. 3A). Stride time, defined as the time elapsed between two successive initiations of stances, was also significantly increased for hSOD1 males compared to WT controls (Fig. 3B). Interestingly, female hSOD1 mice did not exhibit slowing of running speed (Fig. 3C) nor an increase in stride time (Fig. 3D), even at 7 months of age. For the male hSOD1 cohort, there were feeding group differences in average running speed (Fig. 3E) over time by repeated-measures ANOVA (*p* = 0.01) with no *post-hoc* differences. There was no effect of bacterial feeding on stride time (Fig. 3F). However, curli-fed mice displayed significant maximum lateral deviation of hind feet from the axis of the body (Fig. 3G), inability to efficiently move the body from a straight axis (rotation) (Fig. 3H) and a smaller paw print area of the hind feet (Fig. 3I) at 6 and 7 months, as indicated. No significant locomotive abnormalities on any measures were detected in female hSOD1 mice by TreadScan analyses.

Overall, curli-fed hSOD1 males showed significant maximum lateral deviation of hind feet and variations in rotation compared to vehicle and mutant-fed hSOD1 males measured with TreadScan analyses. These measures of locomotion have not been associated previously with the ALS phenotype in mice.

### Chronic gut exposure to mutant and curli-producing *E. coli* enhanced markers of skeletal muscle atrophy

**Figure 4:**
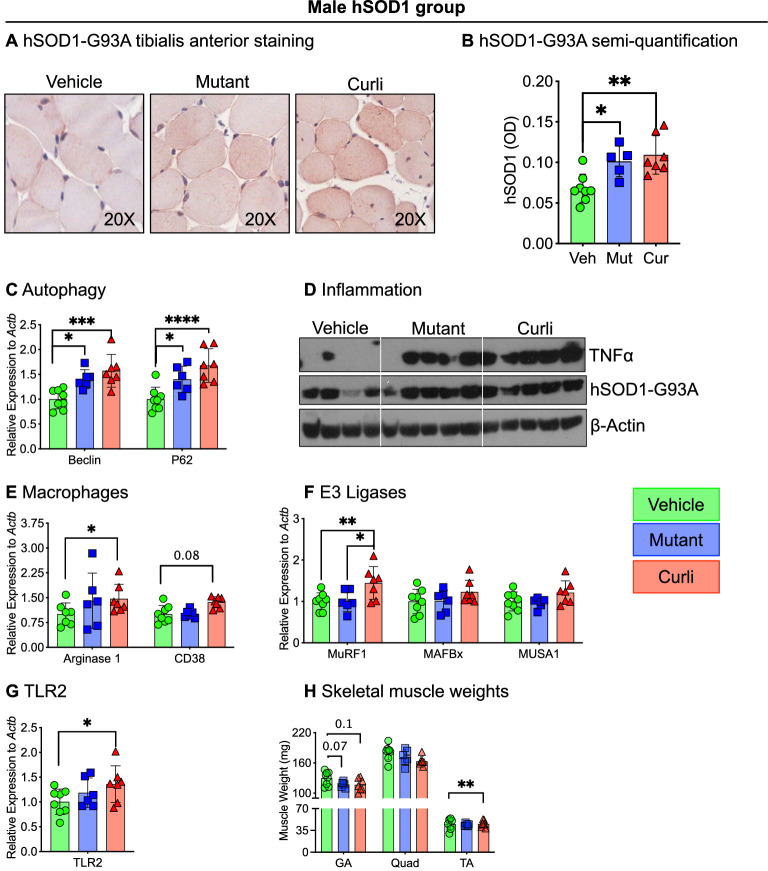
Markers of skeletal muscle atrophy by histochemical staining (tibialis anterior), Western blot analysis (gastrocnemius) and qRT-PCR (gastrocnemius) in the male hSOD1 group. (**A**,**B**) Mutant and curli-fed groups showed significantly increased immunostaining for hSOD1 in tibialis anterior muscles. Images were quantified in ImageJ utilizing optical density for positive staining. (**C**) qRT-PCR analysis demonstrated significantly increased expression of autophagy markers, Beclin and p62 mRNA in mutant and curli-fed mice compared to vehicle. (**D**) Western blot analysis showed increased expression of pro-inflammatory TNFα protein in mutant and curli-fed groups compared to vehicle. (**E**) Inflammatory macrophage populations M1 (CD38) and M2 (Arginase 1), were both significantly elevated in gastrocnemius of curli-fed mice compared to vehicle. (**F**) mRNA expression of an E3 ligase MurF1 was increased 1.5-fold in curli-fed male hSOD1 mice compared to mutant and vehicle groups. (**G**) mRNA expression of TLR2, a pathogen recognition receptor was significantly increased 1.4-fold in curli-fed group compared to vehicle. (**H**) Skeletal muscle weights of gastrocnemius (GA), quadriceps (Quad) and tibialis anterior (TA) were measured post-mortem and curli-fed mice had significantly lower weights of the tibialis anterior muscles compared to vehicle and mutant-fed groups. *p < 0.05, **p < 0.01, ***p < 0.001, one or two-way ANOVA. n = 6–8 per group.

We examined tibialis anterior for histochemical staining and gastrocnemius for mRNA and protein expression for several markers of muscle atrophy. We performed immunohistochemistry in the tibialis anterior muscle utilizing an antibody specific for hSOD1-G93A protein (MS785, GTX57211). Within the male hSOD1 cohort, both mutant and curli-fed mice showed increased hSOD1-positive staining compared to vehicle (Fig. 4A,B). Increased hSOD1-G93A staining may indicate increased SOD1-positive aggregations, but we did not specifically test for aggregations. In gastrocnemius, Beclin and p62, markers of autophagy, were significantly increased in both mutant and curli-fed groups not only in hSOD1 males (Fig. 4C) but in all cohorts—male WT, female WT and female hSOD1 (Supp. Fig. 4A–C). TNFα, a well-known muscle cachectin, was significantly increased in gastrocnemius of both bacterial groups in hSOD1 males (Fig. 4D) as well as in the other 3 cohorts (Supp. Fig. 4D–F). Transcriptional expression of Arginase 1 (*p* < 0.05) and CD38 (trending), both markers of activated macrophages were elevated in curli-fed groups in hSOD1 males (Fig. 4E). Curli-fed groups demonstrated increased mRNA expression of E3 ligases such as MurF1 in hSOD1 males (Fig. 4F), hSOD1 females (Supp. Fig. 4J) and WT females (Supp. Fig. 4L). Taken together, these data indicate increased tissue inflammation and muscle turnover in both mutant and curli-fed groups. Transcription of toll-like receptor 2 (TLR2), a pathogen recognition receptor was increased 1.4-fold in muscle in the curli-fed group compared to vehicle (Fig. 4G), and not statistically significant in other groups (data not shown). Concurrent with increased markers of muscle atrophy in gastrocnemius, mutant and curli-fed male hSOD1 mice had lower weights of the this muscle in hSOD1 males compared to vehicle (trending) (Fig. 4H). Weights of tibialis anterior muscle were significantly lower in curli-fed hSOD1 males compared to vehicle. Bacterial fed hSOD1 females also demonstrated lower skeletal muscle weights (Supp. Fig. 4M).

Overall, chronic feeding of *E. coli*, both mutant and curli-producing *E. coli* increased several markers of muscle atrophy—increased immunohistochemical staining for hSOD1-G93A, upregulation in expression of autophagy markers, inflammatory TNFα, activated macrophages, E3 ligases, TLR2 and lower weights of skeletal muscle in of male hSOD1 mice. Several of these markers were also upregulated in bacterial-fed female hSOD1 mice.

### Chronic gut exposure to mutant and curli-producing *E. coli* increased markers of astrogliosis in the brain and demyelination in the spinal cord

**Figure 5:**
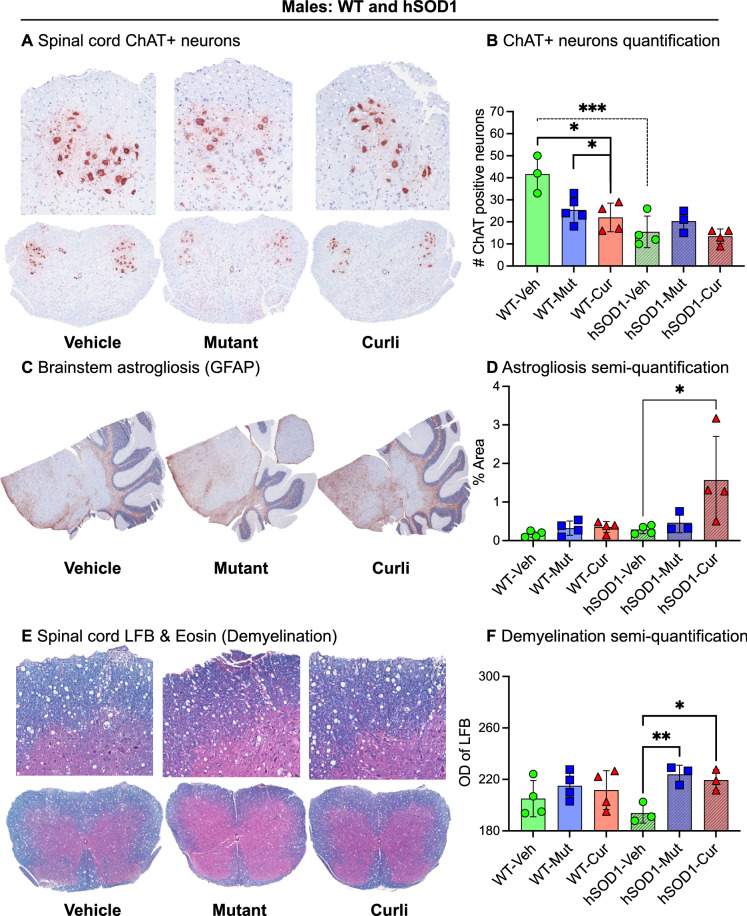
Neurodegeneration, inflammation, and demyelination in the nervous system. (**A**,**B**) Within the male WT group, mutant and curli-fed mice showed significantly decreased cholinergic (ChAT+) neurons compared to vehicle. Within the vehicle group, hSOD1 males had fewer ChAT+ neurons compared to WT controls. (**C**,**D**) Within the male hSOD1 cohort, curli-fed mice showed increased astrogliosis in brainstem compared to vehicle. Images were quantified in ImageJ utilizing percent positive signal of staining. (**E**,**F**) Within the male hSOD1 cohort, mutant and curli-fed mice showed significant demyelination in white matter of spinal cords compared to vehicle groups (encroachment of pink cytoplasmic stain eosin into areas of Luxol Fast Blue stained myelin). *p < 0.05, **p < 0.01, ***p < 0.001, one-way ANOVA. n = 3–5 per group.

As expected in this lower motor neuron degenerative disease, cholinergic neurons detected by choline acetyltransferase (ChAT) immunostaining were significantly decreased in spinal cords of all hSOD1 males, regardless of feeding groups, compared to WT males (Fig. 5A,B). Interestingly, among WT male mice, bacterial exposure to mutant and curli-producing *E. coli* showed significant reduction in ChAT positive neurons compared to vehicle (Fig. 5B). There were no significant differences in ChAT staining in females by genotype or feeding groups (Supp. Fig. 5A). Non-neuronal cells such as astrocytes and microglia can exacerbate ALS progression^35^ and were significantly increased in the lumbar spinal cords of hSOD1 mice compared to WT controls in both males and females with no effect of bacterial feeding in any group (Supp. Fig. 5B–E). However, curli-fed male hSOD1 mice demonstrated significant astrogliosis (glial fibrillary acidic protein, GFAP+) in brainstems compared to mutant and vehicle groups (*p* = 0.03) (Fig. 5C,D). Mice exposed to both bacterial groups showed significantly more demyelinated white matter in the lumbar spinal cord as compared to the vehicle group (*p* = 0.006), measured with Luxol Fast Blue stain (Fig. 5E,F). This difference in demyelination was not detected in females (Supp. Fig. 5F).

Overall, spinal cords of mutant and curli-fed mice showed increased signs of demyelination in hSOD1 males. Curli-fed hSOD1 males also demonstrated increased astrogliosis in the brainstem.

### Chronic gut exposure to curli-producing *E. coli* suppressed peripheral immune responses in hSOD1 male mice

**Figure 6:**
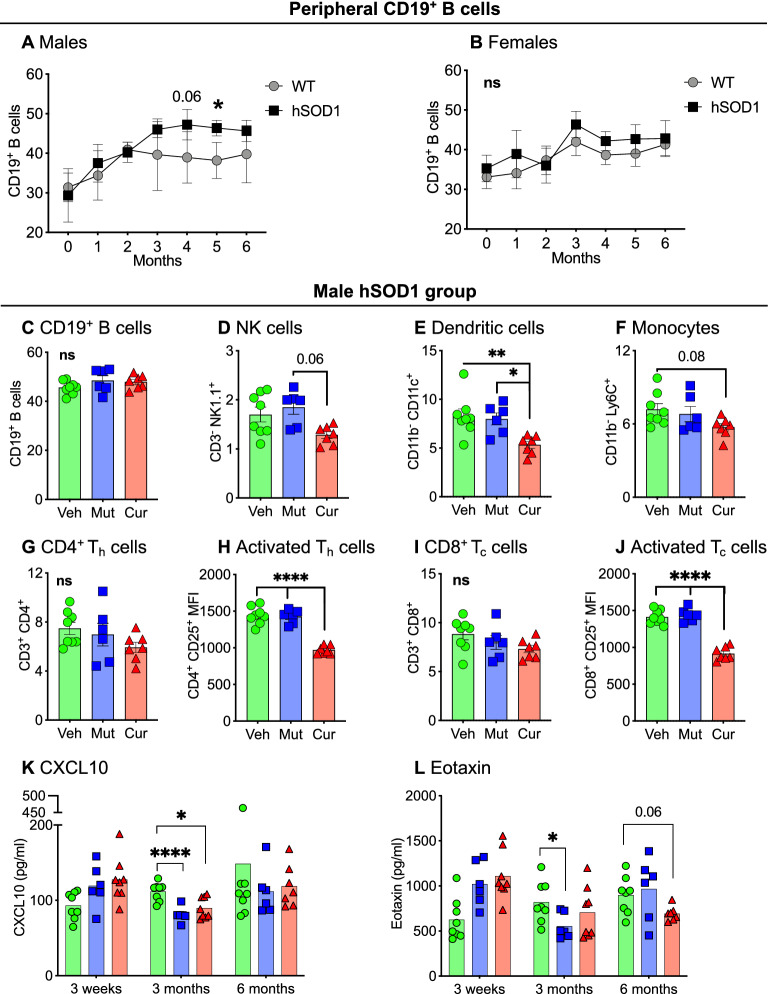
Peripheral blood immune responses. (**A**) Immunophenotyping of peripheral blood demonstrated significantly increased CD19^+^ B cells in hSOD1 males compared to WT controls, month 4 onwards, This phenomenon was not observed in female mice (**B**), mixed-effects model analyses (REML). (**C**–**J**) Within the male hSOD1 cohort, curli-fed mice exhibited significantly decreased markers of NK cells, dendritic cells, monocytes, activated T_h_ cells and activated T_C_ cells. (**K**, **L**) Pro-inflammatory cytokines CXCL10 (3 months) and eotaxin (6 months, trending) were decreased in both bacterial-fed male hSOD1 mice compared to vehicle groups. ns = not significant, *p < 0.05, **p < 0.01, ****p < 0.001, one- or two-way ANOVA, n = 6–8 per group.

We examined monthly changes in circulating immune cell populations and cytokines in peripheral blood. The most striking finding was the significant increase of B cells (CD3^−^ CD19^+^) in male hSOD1 mice compared to male WT mice, month 4 onwards (Fig. 6A). Female hSOD1 mice did not demonstrate this increase (Fig. 6B). Bacterial feeding did not influence peripheral B cell population (Fig. 6C, Supp. Fig. 6A,B). In hSOD1 male (Fig. 6D–J) and WT male (Supp. Fig. 6C–K) cohorts, curli-fed mice showed significantly decreased expression of several innate immunity markers such as NK cells (CD3^−^ NK1.1, Fig. 6D), plasmacytoid dendritic cells (CD3^−^ CD11b^−^ CD11c^+^, Fig. 6E), and monocytes (CD3^−^ CD11b^−^ Ly6c^+^, Fig. 6F). In the adaptive immune arm, curli-exposed animals had significantly decreased expression of CD4^+^CD25^+^ activated T_h_ cells (Fig. 6H) with no difference in CD4^+^ T_h_ cell frequency (Fig. 6G). Similarly, there was significantly decreased expression of activated CD8^+^ CD25^+^ T_C_ cells in curli-exposed hSOD1 males (Fig. 6J), with no significant differences in cytotoxic T_C_ cell frequency (CD8^+^ T cells, Fig. 6I). Expression of peripheral blood cytokines, CXCL10 (Fig. 6K) at 3 months and eotaxin (Fig. 6L, trending *p* = 0.06) at 6 months, which play a crucial role in recruitment of T cells into sites of tissue inflammation^36–39^ were also decreased in curli-fed hSOD1 males. In female WT and hSOD1 mice, both mutant and curli-producing *E. coli* groups showed significant suppression of the above innate and adaptive immune markers (Supp. Fig. 6C–K).

Overall, chronic feeding of curli producing *E. coli* led to suppressed peripheral immune responses in all males, whereas both mutant and curli producing *E. coli* led to suppressed peripheral immune responses in all females.

### Chronic gut exposure to *E. coli* did not alter gut permeability or whole-gut transit time at 6 months of age

**Figure 7:**
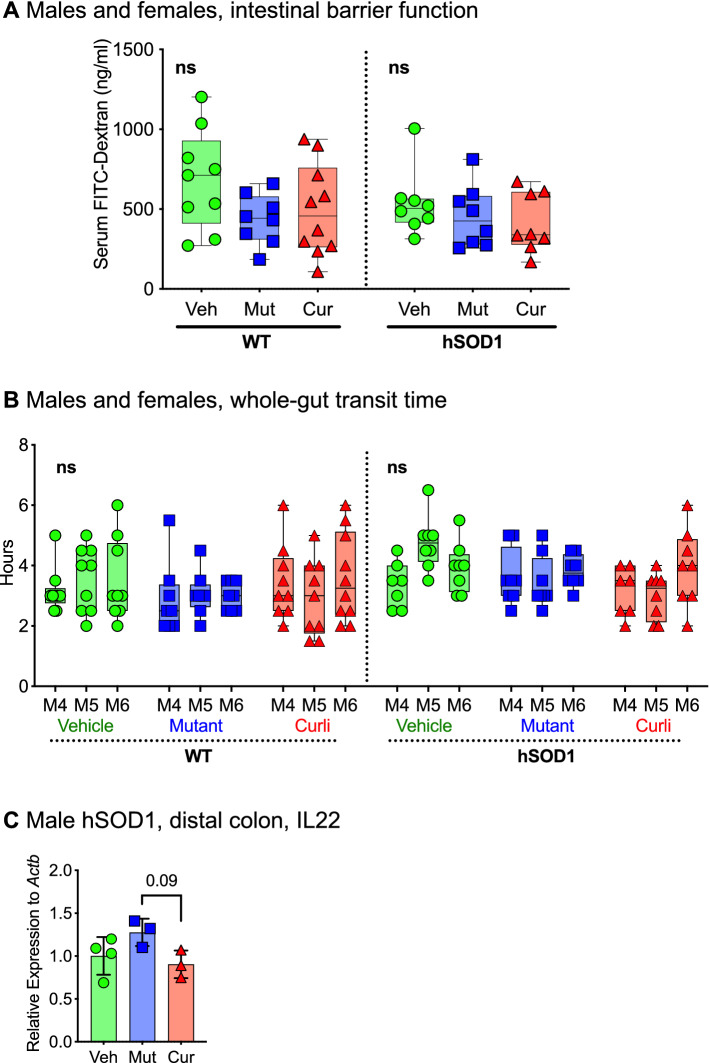
Functional characteristics of the gastrointestinal system (**A**) Gut barrier integrity was assessed by oral administration of FITC-Dextran and measuring its absorption in circulating blood 4 h later. No differences in serum FITC signal was detected between any groups. n = 8–10 per feeding group, males and females, randomly selected for the assay, one-way ANOVA. (**B**) There were no significant differences in whole-gut transit time at months 4, 5, and 6 between genotypes, sex or bacterial feeding groups measured as time taken (hours) to excrete carmine red dye administered orally. n = 8–10 per feeding group, males and females, randomly selected for the assay, one-way ANOVA. (**C**) Within the male hSOD1 cohort, mRNA expression of IL22 was decreased (trending) in curli-fed male hSOD1 mice compared to the mutant group, Kruskal–Wallis test, n = 3–4 per group.

We measured gut barrier function in live mice at 6 months with oral administration of FITC-dextran, followed by quantification of FITC signal in serum 4 h later. There were no significant differences in serum FITC signal between WT and hSOD1 mice, indicating absence of a gut barrier breach at this stage. Additionally, there were no differences in serum FITC signal between bacterial-fed groups and vehicle for any mice (Fig. 7A). There were no significant changes in whole-gut transit time at 4, 5 and 6 months measured as time taken (hours) for the appearance of a red pellet after oral administration of carmine red dye, regardless of genotype or bacterial feeding (Fig. 7B). IL22, produced by activated T cells were slightly decreased (trending) in distal colon of curli-exposed male hSOD1 mice (0.9-fold vs. mutant) (Fig. 7C). IL22 plays a role in potentiating pro-inflammatory responses^40^.

Overall, there were no indications of increased gut permeability in hSOD1-G93A mice or in any bacterial fed groups at 6 months of age.

## Discussion

Intestinal dysbiosis is commonly associated with gastrointestinal diseases and there is increasing evidence that microbiome composition may influence development of neurodegenerative disorders such as Parkinson’s disease^7,28,29^, ALS^15–17,41^ and Alzheimer’s disease^11,13^, neurodevelopmental diseases such as autism spectrum disorder^18,19^, and depression^42^. Recently, it was reported that there is significant expansion of the *E. coli* population in the gut of ALS patients compared to healthy controls^16,17^. Bacterial overgrowth in the intestine is found in approximately 20% of healthy older adults, manifested by mild symptoms such as malabsorption^21^ but dysbiosis can be clinically silent. The role of chronic dysbiosis has not been extensively studied in ALS.

We produced chronic dysbiosis in a mouse model of ALS that had a standard microbiome for its strain and housing facility. Exogenous introduction of a commensal bacteria such as curli-producing *E. coli* shifted the gut microbial composition to induce bacterial and viral dysbiosis in these mice (Fig. 2 and Supp. Figs. 2 and 3). *Escherichia coli* usually inhabit the small intestine and belong to the gammaproteobacterial class of the phylum *Proteobacteria* and are typically associated with inflammatory conditions such as obesity^43^ and inflammatory bowel disease^44^. Consumption of curli-producing *E. coli* significantly inhibited proliferation of *Proteobacterial* species in the distal colon (Fig. 2J–L). We did not detect differences in abundance of *Akkermansia muciniphila* as reported by Blacher et al. in their ALS model^15^. Although these data demonstrated several microbial perturbations in response to bacterial feeding (Fig. 2, Supp. Figs. 2 and 3), there were no outward signs of illness in mice assessed every week with body weight, general appearance, grooming habits, posture, and interaction with cage mates. Measures of locomotion and peripheral inflammation were evaluated every month, discussed later.

We examined skeletal muscles of mice for several markers of inflammation and atrophy (Fig. 4). Mice exposed to bacteria (both, *E. coli-*curli and mutant) exhibited increased TNFα protein expression in gastrocnemius, suggesting presence of inflammation in skeletal muscle. In subjects with familial and sporadic ALS, skeletal muscles harbor impairment in protein quality control processes such as autophagy^45,46^ and proteasomal degradation^47–51^. Autophagy is upregulated in ALS secondary to accumulation of insoluble protein aggregates, stress granules, and damaged mitochondria. Expression of E3 ubiquitin ligases such as Murf1, Mafbx (Atrogin1) and Musa1 target proteins for degradation and positively correlate with muscular atrophy^47,51^. Curli-fed mice displayed significantly higher mRNA expression of Beclin, p62 (autophagy markers) and Murf1 and significantly lower weights of the tibialis anterior and gastrocnemius muscles. Interestingly, all groups of mice fed *E. coli*, both mutant and curli-producing, demonstrated an increase in several of these atrophic markers in skeletal muscles (Supp. Fig. 4).

We assessed the lumbar spinal cord and brainstems for markers of neuronal inflammation and a decrease in cholinergic neurons (lower motor neuron degeneration), which accompany skeletal muscle atrophy in ALS. Curli-exposed hSOD1 male mice exhibited significantly increased astrogliosis in the brainstem as seen with our study in rats^29^. Both *E. coli*-fed groups showed significant demyelination in the lumbar spinal cord. In terms of lower motor neuron (LMN) degeneration measured with choline acetyltransferase staining (ChAT, Fig. 5A,B), hSOD1 male mice showed a significant decrease in the number of cholinergic neurons in the spinal cord, compared to WT mice. There were no additional effects of bacterial feeding on cholinergic neuron numbers. In our study, markers of muscle atrophy were most enhanced in curli-exposed hSOD1 male mice even though there was not more LMN degeneration in this group compared to vehicle or mutant fed *E. coli* animals. LMN degeneration, which is characteristic of ALS may not be the only preceding event to muscle degeneration and may occur independently, simultaneously or in reaction to muscle atrophy^52^. A skeletal muscle only knock-in model of the hSOD1-G93A transgene could develop an ALS phenotype in mice^53^, suggesting that skeletal muscle can develop its own pathology independent of LMN degeneration.

One of the most important findings of this study was the role of peripheral immune system in this ALS mouse model. In the vehicle groups absent of bacterial feeding, hSOD1 male mice displayed significantly increased frequency of CD19^+^ B cells, month 4 onwards compared to WT males, which did not occur in female mice. We hypothesize that this phenomenon may at least partially contribute to an earlier onset of ALS in males and warrants investigation. At 7 months of age, within the male hSOD1 (Fig. 4) and male WT (Supp. Fig. 4) groups; expression of circulating innate immune cells such as natural killer NK cells (CD3^-^ NK1.1^+^), dendritic cells (CD11b^-^ CD11c^+^) and monocytes (CD11b^+^ Ly6c^+^) were significantly decreased in peripheral blood of *E. coli* curli-fed males. Expression of circulating adaptive immune cells such as activated T_h_ cells (CD4^+^CD25^+^), activated T_C_ cells (CD8^+^CD25^+^) were also significantly decreased in the peripheral blood of curli exposed males. T-cell chemoattractant plasma cytokines such as CXCL10 and eotaxin were also decreased at 3 and 6 months respectively within the hSOD1 males. Several published reports of ALS patients revealed that expression of CD4^+^CD25^+^ T cells correlate inversely with rapid ALS progression^54–56^, similar to our findings in curli-fed hSOD1 males. While curli-fed mice had dampened peripheral immune responses in male hSOD1 mice, inflammation at the skeletal muscle level was increased as evidenced by increased TNFα protein expression in gastrocnemius muscle. Since curli proteins exhibit high affinity for MHC1 receptors^57^, we hypothesize that association between curli and MHC1 can enhance adhesion and colonization of *E. coli* in the gut. Such an interaction can potentially interfere with the antigen presenting function of MHC1 molecules to cytotoxic T cells (T_C_). This phenomenon may contribute to suppressed peripheral immune responses in mice and warrants experimentation.

The role of peripheral inflammation in ALS is poorly understood, with conflicting reports of favorable and adverse roles in disease progression^58^. Our data highlight of the role of increased frequency of peripheral CD19^+^ B cell population in male hSOD1 mice which were accompanied by suppression of critical innate and T cell responses in curli-fed males. However, female hSOD1 groups also demonstrated similar peripheral immune suppression in response to both curli-producing and non-producing *E. coli* (Supp. Fig. 4), but expansion of CD19^+^ B cells were absent. Female hSOD1 mice did not demonstrate signs of locomotive impairment with TreadScan analysis (Fig. 3). The immune suppression theory is also postulated by the Appel research group^54,56,59^.

To summarize the important findings, we examined the role of *E. coli* induced dysbiosis in progression of ALS and found sex-specific effects in our ALS mouse model. Male hSOD1 mice demonstrated significantly poorer microbial species diversity. Almost all cohorts showed decreased abundance of the *Proteobacteria* phylum. Male mice showed suppressed peripheral immune activation in response to *E. coli* curli, while females demonstrated suppressed peripheral immune activation in response to both curli-producing and non-producing *E. coli*. Male hSOD1 mice demonstrated gradual slowing in running speed month 4 onwards, while females exhibited no signs of locomotive impairment even at 7 months of age, measured with TreadScan. Both mutant and curli-producing *E. coli* exposed groups demonstrated several markers of skeletal muscle pathology—higher transcriptional expression of autophagy markers, E3 ligases and pro-inflammatory macrophages; increased protein expression of TNFα, lower weights of skeletal muscles and increased hSOD1 immunostaining in tibialis anterior muscles specifically within the male hSOD1 group. Curli-fed male hSOD1 mice also demonstrated increased astrogliosis in brainstem. Skeletal muscles demonstrated 1.4-fold higher mRNA expression of toll-like receptor 2 (TLR2), a pathogen recognition receptor, compared to vehicle controls. We have previously shown that bacterial curli-exposed aged rats had higher TLDR2 expression in striatum and hippocampus regions of the brain^29^.

We were unable to assess how long-term *E. coli* exposure in the gut influences pathology in distant organs. We did not test for presence of *E. coli* or curli protein in peripheral blood, skeletal muscle, spinal cord, or brain. We did not detect a breach in the gastrointestinal barrier in mice at 6 months measured with FITC-dextran administration. A recent report showed that FLAG-tagged curli produced by *E. coli* fed to *C. elegans* was able to colocalize with α-synuclein in neurons^27^ and curli protein uptaken by human neuroblastoma cell lines enabled cross-seeding of SOD1-G85R protein in *C. elegans*^27^. This opens the possibility of transport of curli and other microbial products from gut to distant organ systems and is of tremendous value to the field of non-familial neurodegeneration.

In conclusion, chronic exposure to curli-producing and non-producing *E. coli* exacerbated certain features of ALS. Some of these features were more striking in curli-exposed animals. Our data suggest that increased peripheral blood frequency of CD19^+^ B cells may contribute to earlier onset of motor impairment in males. We agree with Burberry et al.^60^ and Figueroa-Romero et al.^61^ that microbial influences outside of the nervous system are involved in ALS.

### Strengths and limitations

Some of our data may differ from published studies of hSOD1-G93A mice that utilized a faster progression model. One of the most important limitations of this study was the inability to assess the influence of *E. coli* induced gut dysbiosis on full-blown hindlimb paralysis and survival. Currently, we lack the tools and techniques to potentially detect curli protein in distant organs. Some of the strengths include our mouse model and study design. We utilized a slower onset of ALS mouse model to enable early investigation of subtle motor impairments as well as changes in peripheral inflammation. Chronic gut dysbiosis was developed by a common commensal bacteria like *E. coli* with curli-lacking controls. We evaluated the influence of expansion of *E. coli* in the gut in presence of the mouse’s standard microbiome for strain and facility. As shown by Sampson et al.^28^, polyphenols which inhibit bacterial amyloid aggregation in the gut in a mouse model of Parkinson's disease may be worth investigating in ALS.

## Methods

### Animals

Male and female hSOD1-G93A mice were purchased from The Jackson Laboratory (JAX 002,299)^31^. C57BL/6J-congenic SOD1-G93A transgenic mice carry a reduced copy number of the variant human superoxide dismutase 1 soluble gene transgene, Tg(SOD1*G93A)^dl^1Gur. This slower ALS hSOD1-G93A strain develops the motor phenotype at six to seven months of age. Male Tg(SOD1*G93A)^dl^1Gur founder mice were bred with C57BL/6J wild-type control mice. Transgenic mice and their littermate controls were housed in conventional, autoclaved ventilated caging.

All animal husbandry and experimental protocols were approved by the University of Louisville’s Institutional Animal Care and Use Committee (IACUC) ethics committee. All methods were carried out in accordance with relevant guidelines and regulations. All methods are reported in accordance with ARRIVE guidelines.

### Bacterial strains and characterizations

*Escherichia coli* strain BW25113 (WT) was first transformed with an empty plasmid containing an ampicillin resistance cassette (pET15b) as our selection marker and genotyped to confirm the presence of curli operon. *Escherichia coli* strain BW25113 csgGFED_BAC::FRT-kan-FRT (curli -) harbored a kanamycin resistance cassette and was genotyped to confirm knockout of the curli operon. These strains were cultured aerobically in LB media supplemented with each respective antibiotic marker at 37 °C overnight. Ampicillin and kanamycin LB agar plates were inoculated and left to grow at room temperature to further induce curli expression, confirmed via Western Blot analysis (data not shown). Plates were scraped and the bacterial strains were resuspended for feeding as described below.

### Bacterial preparation for animal feeding

The indicated bacterial strains were grown fresh for each feeding session and collected immediately prior to dosing. The bacterial strains were resuspended in 1.5% sodium bicarbonate in PBS (Phosphate Buffered Saline) and then mixed with the applesauce. The animals received ~ 10^9^ CFU of bacteria per feeding session, three times per week over the course of 6 months. The animals were maintained on regular chow diet when not being fed bacterial strains. Confirmation of bacterial dosing was conducted by collecting fecal pellets and resuspending them in PBS and then plating them on each feeding group’s respective ampicillin (BW25113 WT) or kanamycin (BW25113 Curli-) LB agar plates. Colonies were picked and genotyped as described below. Applesauce consumption was monitored after each feeding session to ensure proper dosing (data not shown). Body weights were monitored every other week and blood was collected from the submandibular vein monthly over the course of the study.

Rather than gavaging the mice three times per week, we provided a less stressful means of delivering bacteria to the mice via applesauce (adapted from Hsiao et al. Cell 2013^62^). The mice were fasted for 2 h prior to feeding to encourage consumption of a novel substance. Upon weaning, all cage mates were acclimated to organic, unsweetened applesauce mixed with 1.5% sodium bicarbonate in PBS. 1 ml of applesauce solution was spread over a food pellet placed in a sterile, micro-petri dish (35 × 10 mm “micro dish”) and placed in an empty autoclaved cage free of bedding and food. After acclimation in a group setting, the mice were separated individually into autoclaved cages free of bedding and food pellets and were fed the applesauce solution similarly. Their individual consumption was monitored for every feeding day as percent of food consumed by trained observers. The mice tolerated the applesauce well and would typically consume all of it over the course of 4–6 h.

### Motor function assessment

For all motor assessments, each test utilized the same handlers, same blinded examiner and were conducted at the same time every month (3 weeks–7 months). After each mouse, the assessment area was sprayed with 70% ethanol and wiped down. To reduce the odds of bacterial intermingling between groups, the three groups were run separately. Motor function were assessed using the following instruments: TreadScan and Basso Mouse Scale.

### Motor function assessment—Treadmill Gait Kinematics (TreadScan)

Mice walking on a treadmill were recorded from below using a camera and TreadScan software as described previously^34^. Mice were first placed onto the treadmill and allowed to investigate the area for ~ 20 s to relax and get accustomed to the area. The treadmill is then turned on and the speed is slowly ramped up to allow the animals to get used to the task. The animals are then recorded for 2000 frames which is no more than 1 min. These videos allow analysis of the following 37 gait characteristics as well as regularity index and plantar stepping index: stride time, stance length, stance length, stance time, swing time. brake time, propulsion time, percentage of stance, percentage of swing, stride length, average print area, max lateral deviation, minimum lateral deviation, mac longitudinal deviation, minimum longitudinal deviation, front track width, rear track width, left foot base, right foot base, instantaneous running speed, average running speed, overall running speed, absolute stride number, normalized stride number (stride frequency), homologous coupling, homolateral coupling, diagonal coupling, sciatic function index print length, sciatic function index toe spread, sciatic function index intermediary toe spread, sciatic function index print angle, gait angle, body rotation average, body rotation standard deviation, longitudinal movement average, lateral movement average, longitudinal movement standard deviation, lateral movement standard deviation, ratio index, coordinated pattern index and plantar stepping index. Repeated measures two-way ANOVA or mixed-effects model analyses (REML) with Tukey’s multiple comparisons. n = 6–8 per group.

### Intestinal permeability assay

FITC-dextran allows for the assessment of intestinal permeability and potential leaky gut. FITC-dextran (MW 4000, Millipore-Sigma) was dissolved in PBS to yield a 60 mg/ml dosing solution concentration. Animals were fasted for 4 h and then administered the FITC-dextran solution by oral gavage at a dose volume of 10 ml/kg. The animals were returned to their cages for 4 h and then blood was collected from the submandibular vein. Serum was isolated, diluted, and measured with a plate reader (Ex/Em 485/530) Values were interpolated from a standard curve. n = 8–10 per feeding group, males and females, randomly selected for the assay, one-way ANOVA.

### Whole-gut transit time

This test allows assessment of enteric nervous system or colonic abnormalities as described^63^. A 6% w/v carmine red (Millipore-Sigma) solution in 0.5% methylcellulose was produced. A dose volume of 10 ml/kg was used, and the solution was administered by oral gavage. After dosing, mice were moved to individual cages with white bedding and monitored every 30 min for the appearance of the first red pellet for up to 8 h post-dose. Times for each group were recorded, averaged, and compared for delay in whole-gut transit time between bacterial feeding groups. n = 8–10 per feeding group, males and females, randomly selected for the assay, one-way ANOVA.

### ALS pathology and inflammatory responses

Animals were anesthetized with isoflurane following institutional IACUC regulations and perfused with cold PBS and various tissues relevant to ALS were collected including skeletal muscle from hind limbs, spinal cord, distal colon, and brain. Skeletal muscle wet weights were recorded.

### qRT-PCR

The tibialis anterior (TA) muscle and distal colon were homogenized, and RNA was extracted using an EZNA Total RNA kit (Omega Bio-Tek) according to manufacturer instructions. cDNA was made using high-capacity reverse transcription kit (Applied Biosystems) and qPCR was run on a CFX96 Real Time PCR system (BioRad) using SybrGreen master mix (Applied Biosystems). Primers utilized include the following: *Beta Actin, TNFα, IL1b, MuRF1, Beclin, MAFBx, MUSA1, P62, Arginase1, , iNOS, CD38, TLR2, ZO-1, IL22.* Please see the table for primer sequences. Results are reported using the ΔΔCT method.

### Western blot analysis

The tibialis anterior muscle, distal colon, and brain were homogenized in RIPA buffer and protein concentrations were measured using BCA (bicinchoninic acid) assay (Pierce 23,225). The following antibodies were used hSOD1-G93A (SOD1 MS785, GTX57211, Genetex), TNFα (ABclonal A0277) and β-actin (ABclonal AC026).

### Immunohistochemistry

The tibialis anterior muscle, distal colon, lumbar spinal cord, and brain were post-fixed in 10% neutral buffered formalin for 48 h and then switched to 70% ethanol until ready for embedding with paraffin. Paraffin embedded samples were cut into 6-8um sections on slides for staining. Tissue sections were deparaffinized, rehydrated and probed with the following antibodies: hSOD1 (SOD1 MS785, GTX57211), ChAT (ProteinTech, 20,747-1-AP), GFAP (ABclonal, A10873), Iba1 (Abcam, ab17886), Histochemistry was performed using antigen retrieval with citric acid buffer at 95C for 30 min and Vector ABC and NovaRed substrate system according to manufacturer instructions (Vector Laboratories). Please refer to the table for additional antibody information. Luxol Fast Blue (LFB) and eosin stain of the spinal cord were performed by the Dept. of Pathololgy at University of Louisville. Slides were scanned using an Aperio Slide Scanner (Leica Biosystems) with a 20X objective. Images were quantified in ImageJ using optical density, mean gray value, or % area depending on context, described in figure legends.

### Flow cytometry analyses

Flow cytometry analysis were performed every month (3 weeks onwards up to 7 months). Blood was collected (100–200 ul) using a small needle prick in the submandibular vein in EDTA collection tubes. 50ul whole blood was utilized for following steps: incubation with Fc block (10 min, ice) and then immunofluorescent antibodies (30 min, room temp, dark) for the following: CD3 (BD Biosciences 557,724), CD4 (Biolegend 100,406), CD8 (BD Biosciences 564,297), CD19 (BD Biosciences 562,291), NK cells (NK1.1 BD Biosciences 557,391), CD11b (BD Biosciences 563,402), Ly6G (BD Biosciences 561,236), Ly6C (BD Biosciences 560,596), CD11c (BD Biosciences 550,261). RBCs were lysed/fixed (Biolegend 422,401) and post-processing, samples were stored at 4C in dark for 3–4 days, until running on a BD multicolor LSR Fortessa. Data were analyzed using Flow Jo™ 10.5.3. Gating strategy has been provided in Supplementary Fig. 7.

### Cytokine multiplex analysis

Cytokines in the plasma were probed using the cytokine 32-plex using a BioPlex 200 Mouse Cytokine Array (Eve Technologies). Blood samples were spun down in their respective EDTA tubes at 1000×G for 10 min and plasma was collected and stored at − 80 °C until ready to be shipped. 25 ul samples were diluted two-fold in PBS and shipped on dry ice.

### Fecal metagenomics

Fecal pellets were collected and stored at − 80 °C and shipped on dry ice to CosmosID Metagenomics, Rockville, MD. DNA extraction, Illumina library preparation, sequencing at 3 million total reads (1 × 150 bp or 2 × 150) were performed at CosmosID. PCA plots, alpha and beta diversity PCoA were constructed utilizing the Cosmos ID Metagenomics App platform. Relative abundance analyses were performed by our in-house bioinformatician. Statistical tests: Wilcoxon rank sum tests for alpha-diversity, PERMANOVA tests for beta-diversity, Kruskal–Wallis tests for relative abundance comparisons.

### Statistical analysis

Every figure legend and specific sections under Methods provide details on statistical tests performed. Graphs were prepared using GraphPad Prism Version 9.4.1 or Cosmos ID Metagenomics App platform (https://app.cosmosid.com).

### Key resources

Following is a list of key resources and reagents (Table 1).

**Table 1:** Key Resources and Reagents.

Reagent type, species, or resource	Designation	Source or reference	Identifier	Additional info
Strain, strain background (*Mus musculus*)	B6.Cg-Tg(SOD1*G93A)dl1Gur/J, C57BL/6J background	The Jackson Lab	SOD1-G93A	^31^
Strain, strain background (*Escherichia coli*)	Str. BW25113, rrnB3 ΔlacZ4787 hsdR514 Δ(araBAD)567 Δ(rhaBAD)568 rph-1	Matthew R. Chapman	WT (wild-type)	^64^
Strain, strain background (*Escherichia coli*)	Str. BW25113, csgGFED_BAC::FRT-kan-FRT	Matthew R. Chapman	Curli-	^64^
Sequence based reagent	*hSOD1:* 5′-GGGAAGCTGTTGTCCCAAG-3′ And 5′-CAAGGGGAGGTAAAAGAGAGC-3′	Integrated DNA Technologies	gDNA, qPCR Primer	The Jackson Labs
Sequence based reagent	*ApoB*, Internal Positive Control: 5′-CACGTGGGCTCCAGCATT-3′ And 5′-TCACCAGTCATTTCTGCCTTTG-3′	IDT	gDNA, qPCR Primer	The Jackson Labs
Sequence based reagent	*csgA:* 5′-GATCTGACCCAACGTGGCTTCG-3′ And 5′- GATGAGCGGTCGCGTTGTTACC-3′	IDT	gDNA, PCR Primer	
Sequence based reagent	*csgC:* 5′-CCTGTTTTTTTTCGGGAGAAGAATATG-3′ And 5′-ATTCATCTTATGCTCGATATTTCAACAA-3′	IDT	gDNA, PCR Primer	
Sequence based reagent	*csgB:* 5′-ACCAGGTCCAGGGTGACAACATG-3′ And 5′-AGTCGAATGGAAATTAACGTTGTGTC-3′	IDT	gDNA, PCR Primer	
Sequence based reagent	*csgD:* 5′-ACCAGGTCCAGGGTGACAACATG-3′ And 5′-AGTCGAATGGAAATTAACGTTGTGTC-3′	IDT	gDNA, PCR Primer	
Sequence based reagent	*csgE:* 5′-TTTTTATTTAGAATTCATCATGCGCCAA-3′ And 5′-ATAACCTCAGGCGATAAAGCCATG-3′	IDT	gDNA, PCR Primer	
Sequence based reagent	*csgF:* 5′-GGGGCTTAAAAATCGGTTGAGTTATT-3′ And 5′-TAAAAAATTGTTCGGAGGCTGCAATG-3′	IDT	gDNA, PCR Primer	
Sequence based reagent	*csgG:* 5′-TGTCAGGATTCCGGTGGAACCGA-3′ And 5′-CCCAGCTTCATAAGGAAAATAATCATG-3′	IDT	gDNA, PCR Primer	
Sequence based reagent	*ycdZ:* 5′-GAACATACTTCTCTCTATTGCAATCA-3′	IDT	gDNA, PCR Primer	
Sequence based reagent	*ymdA:* 5′-CAAACTGCCGAGCATAAGAGAG-3′	IDT	gDNA, PCR Primer	
Sequence based reagent	*Beta actin (Actb):* 5′-GGCTGTATTCCCCTCCATCG-3′ And 5′-CCAGTTGGTAACAATGCCATGT-3′	IDT	cDNA, PCR Primer	PrimerBank
Sequence based reagent	*MAFBx:* 5′-CAGCTTCGTGAGCGACCTC-3′ And 5′-GGCAGTCGAGAAGTCCAGTC-3′	IDT	cDNA, PCR Primer	PrimerBank
Sequence based reagent	*MuRF1:* 5′-GTGTGAGGTGCCTACTTGCTC-3′ And 5′-GCTCAGTCTTCTGTCCTTGGA-3′	IDT	cDNA, PCR Primer	PrimerBank
Sequence based reagent	*MUSA1:* 5′-TATGAACTGTGTCAGTAGACGGT-3′ And 5′-CGATGTTCGTCAGCTTTACAAGA-3′	IDT	cDNA, PCR Primer	PrimerBank
Sequence based reagent	*Beclin:* 5′-ATGGAGGGGTCTAAGGCGTC-3′ And 5′-TCCTCTCCTGAGTTAGCCTCT-3′	IDT	cDNA, PCR Primer	PrimerBank
Sequence based reagent	*P62:* 5′-AGGATGGGGACTTGGTTGC-3′ And 5′-TCACAGATCACATTGGGGTGC-3′	IDT	cDNA, PCR Primer	PrimerBank
Sequence based reagent	*TNF-alpha:* 5′-CAGGCGGTGCCTATGTCTC-3′ And 5′-CGATCACCCCGAAGTTCAGTAG-3′	IDT	cDNA, PCR Primer	PrimerBank
Sequence based reagent	*IL-1b:* 5′-TTCAGGCAGGCAGTATCACTC-3′ And 5′-GAAGGTCCACGGGAAAGACAC-3′	IDT	cDNA, PCR Primer	PrimerBank
Sequence based reagent	*TLR2:* 5′-GCAAACGCTGTTCTGCTCAG-3′ And 5′-AGGCGTCTCCCTCTATTGTATT-3′	IDT	cDNA, PCR Primer	PrimerBank
Sequence based reagent	*CD38:* 5′-TCCCTCCGTGAGCCATTTTAC-3′ And 5′-CGATGTCGTGCATCACCCA-3′	IDT	cDNA, PCR Primer	PrimerBank
Sequence based reagent	*Arginase-1:* 5′-CTCCAAGCCAAAGTCCTTAGAG-3′ And 5′-AGGAGCTGTCATTAGGGACATC-3′	IDT	cDNA, PCR Primer	PrimerBank
Sequence based reagent	*IL-22:* 5′-ATGAGTTTTTCCCTTATGGGGAC-3′ And 5′-GCTGGAAGTTGGACACCTCAA-3′	IDT	cDNA, PCR Primer	PrimerBank

## Supplementary Information

Supplementary Figures.

## Data Availability

All data generated or analyzed during this study are available from the corresponding author upon reasonable request.
