# Supplementary material for: Mutant and curli-producing E. coli enhance the disease phenotype in a hSOD1-G93A mouse model of ALS
Source: Sci Rep. 2023 Apr 12;13:5945. doi: 10.1038/s41598-023-32594-5 (PMC10097672; doi:10.1038/s41598-023-32594-5)
Supplement: Supplementary file 1 — Supplementary Figures. [file 41598_2023_32594_MOESM1_ESM.docx]

**Supplementary Figures**

**Mutant and curli-producing *E. coli* enhance the disease phenotype in a hSOD1-G93A mouse model of ALS**

Zimple Kurlawala (1) Joseph D. McMillan (2), Richa A. Singhal (3), Johnny Morehouse (4), Darlene A. Burke (4), Sophia M. Sears (5), Eleonara Duregon (6), Levi J. Beverly (7), Leah J. Siskind (5), *Robert P. Friedland (1)

**Supplementary Figures**

1. Applesauce consumption over 6 months
2. Alterations in the viral microbiome
3. List of statistically significant bacterial and viral strains
4. Markers of skeletal muscle pathology in Female hSOD1, Male WT and Female WT groups.
5. Neurodegeneration, inflammation, and demyelination in the nervous system
6. Peripheral immune response in Female hSOD1, Male WT and Female WT groups.
7. Gating strategy for peripheral blood immunophenotyping
8. Full Western Blot film for hSOD1-G93A
9. Full Western Blot film for TNF𝜶
10. Full Western Blot film for β-actin.

Supplementary Figure 1

**Supplementary Figure 1**: Applesauce consumption over 6 months: 1ml applesauce and bacterial solution was dispensed on a 35mm micro dish. Their individual consumption was monitored for every feeding day as percent of food consumed by trained observers. There were no significant differences in consumption of applesauce and bacteria solution between genotype **(A,B)**, sex **(C, D)** or feeding groups **(E-H),** mixed-effects model analyses (REML) with Tukey’s multiple comparisons, **(I)** Copy number of hSOD1-G93A transgene measured with qPCR was not different between males and females, Student’s t-test, ns=not significant.


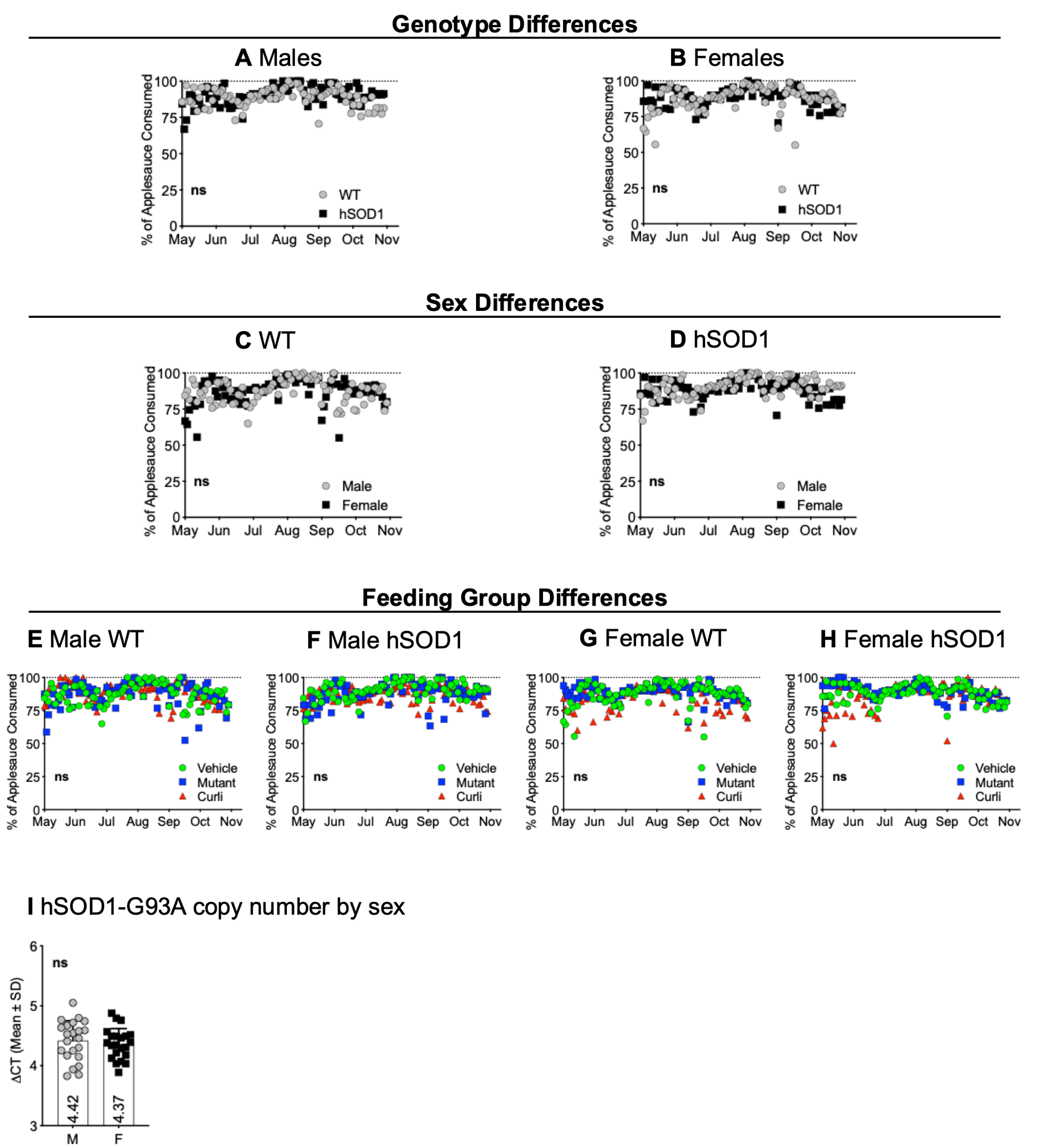


Supplementary Figure 2


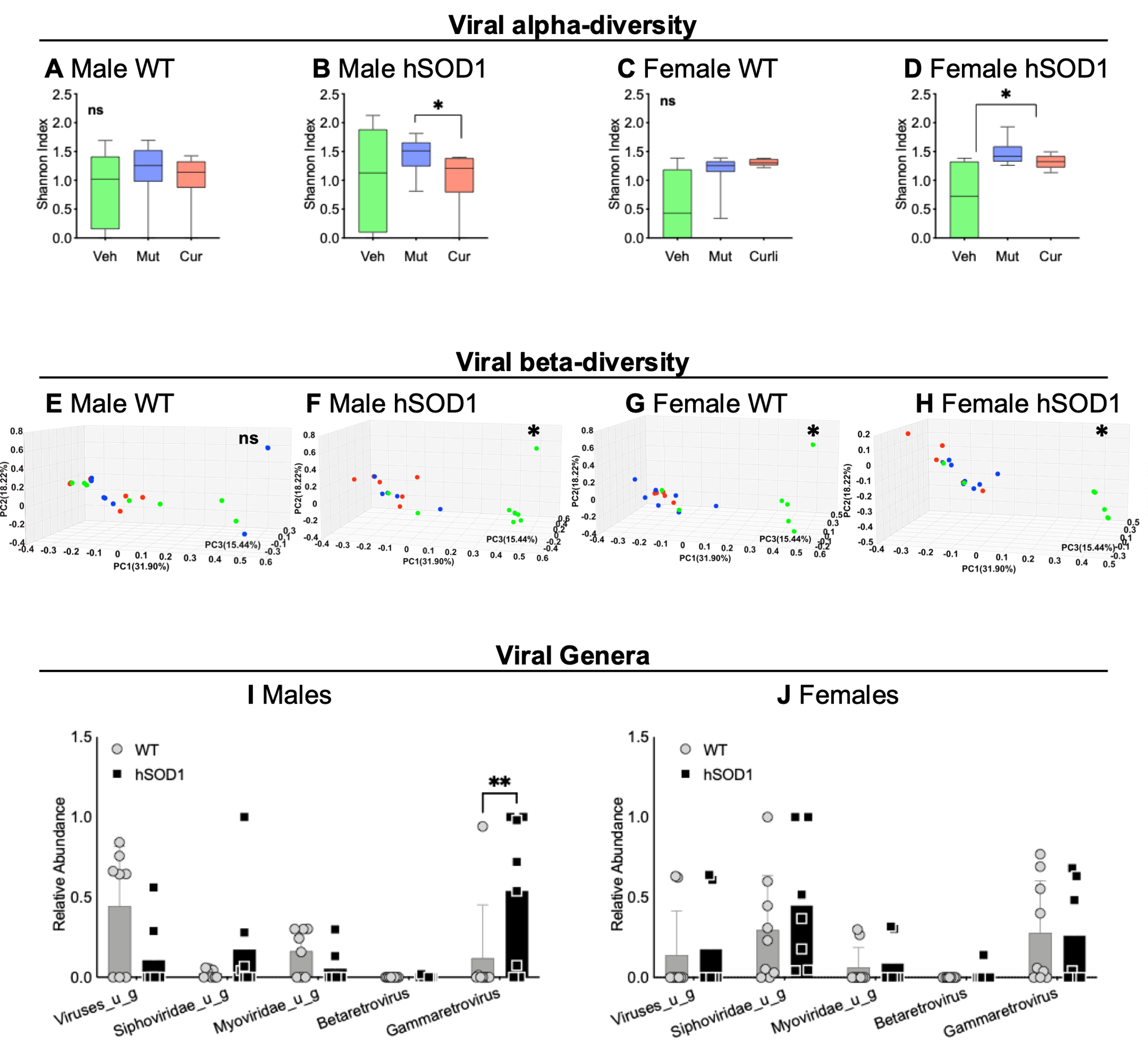


**Supplementary Figure 2:** Alterations in the viral microbiome (**A-D)** Within-group viral species diversity measured with Shannon alpha diversity index *p<0.05, Wilcoxon Rank Sum test. **(E-H)** Between-group viral species diversity measured by JACCARD index demonstrated distinct clustering of the vehicle groups compared to bacterial-fed groups in male hSOD1, female WT and hSOD1 groups. **p<0.01, PERMANOVA test. **(I)** Relative abundance of viral genera in male mice demonstrated significant expansion of gammaretroviruses in hSOD1 mice compared to WT mice. This finding was absent in females **(J),** n=6-9 per group, two-way ANOVA with Šídák's multiple comparisons test.

Supplementary Figure 3

**Supplementary Figure 3:** List of statistically significant bacterial **(A)** and viral strains **(B)** for each cohort acquired by whole genome shallow shotgun sequencing, followed by Kruskal-Wallis analysis. Statistically significant strains at Q-value *p<0.05, **p<0.01 or T(0.05>p<0.1) followed by group comparison (V=Vehicle, M=Mutant, C=Curli) are presented in the table. Data are represented as a heatmap showing mean values of relative abundance for each statistically significant strain. n=6-9 per group.

Supplementary Figure 4

Supplementary Figure 4


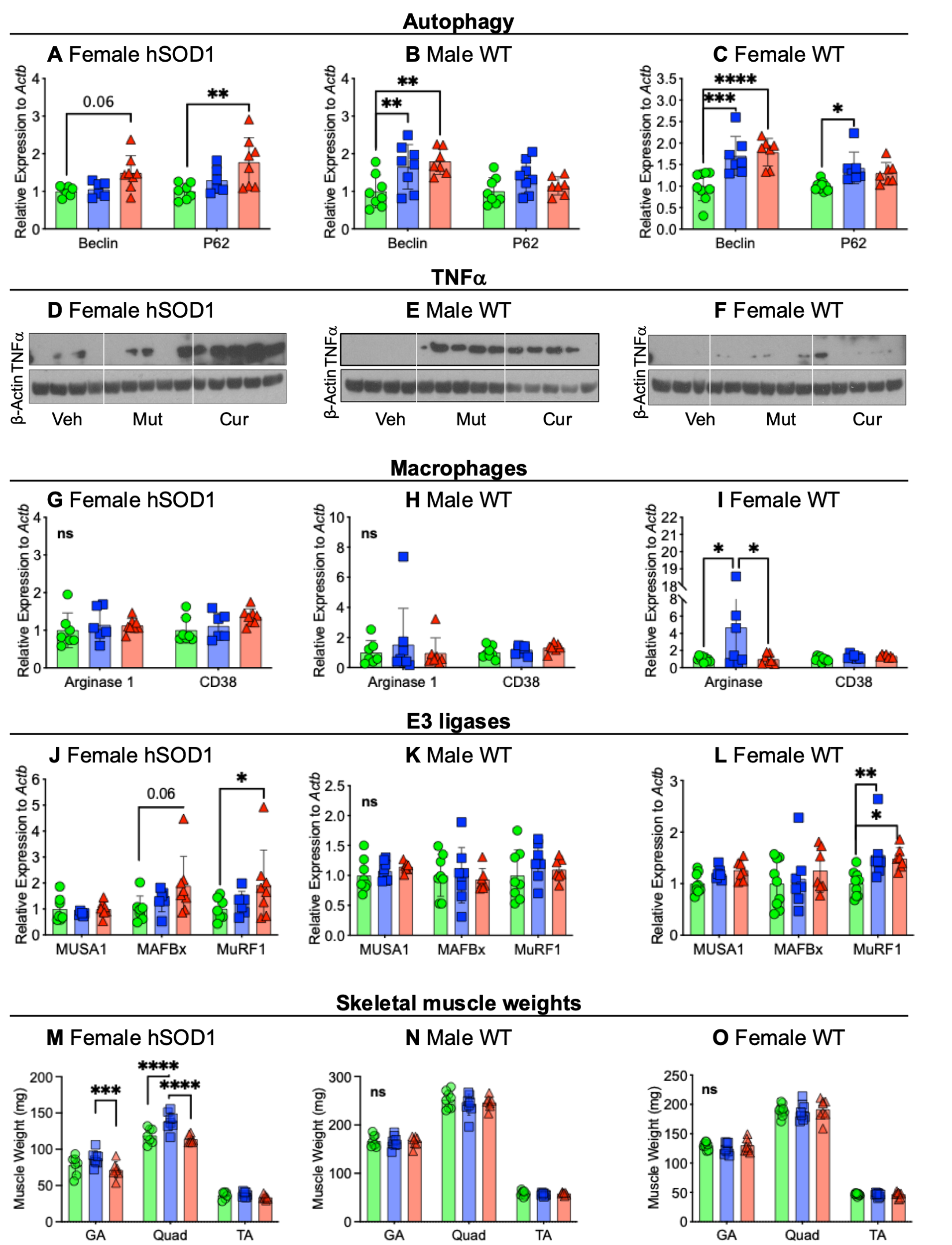


**Supplementary Figure 4**: **(A-C)** qRT-PCR analysis of autophagy markers, Beclin and p62 mRNA in Female hSOD1 **(A)**, Male WT **(B)** and Female WT **(C)** groups. **(D-F)** Western Blot analysis of TNFα protein in in Female hSOD1 **(D)**, Male WT **(E)** and Female WT **(F)** groups. **(G-I)** qRT-PCR analysis of macrophage markers Arginase 1 and CD38 mRNA in Female hSOD1 **(G)**, Male WT **(H)** and Female WT **(I)** groups. **(J-L)** qRT-PCR analysis of E3 ligases MUSA1, MAFBx and MuRF1 mRNA in Female hSOD1 **(J)**, Male WT **(K)** and Female WT **(L)** groups. **(M-O)** Skeletal muscle weights of gastrocnemius (GA), quadriceps (Quad) and tibialis anterior (TA) in Female hSOD1 **(M)**, Male WT **(N)** and Female WT **(O)** groups. ns=not significant, *p<0.05, **p<0.01, ****p<0.001, two-way ANOVA.

Supplementary Figure 5


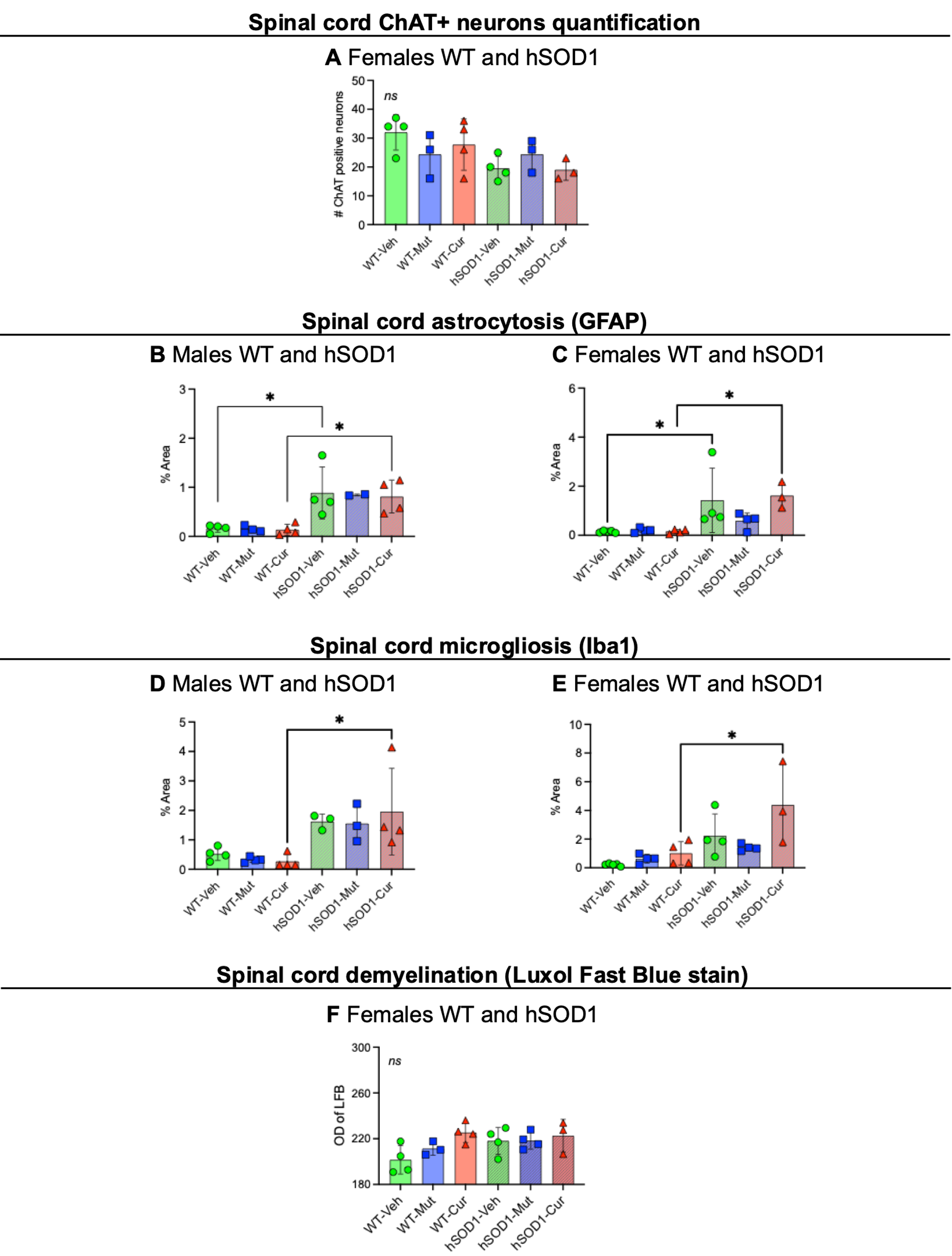


**Supplementary Figure 5**: **(A)** There were no significant differences in quantification of ChAT+ neurons by genotype or feeding groups in spinal cords of female mice**. (B,C**) hSOD1 mice showed increased astrogliosis (GFAP+ staining) in spinal cord compared to WT mice in both males **(B)** and females **(C). (D,E)** Curli-fed mice showed increased microgliosis (Iba1+ staining) in curli-fed hSOD1 mice compared to curli-fed WT controls in both males **(D)** and females **(E). (F)** There were no significant differences in demyelination in white matter of spinal cords in female mice. *p<0.05, one-way ANOVA. n=2-5 per group.

Supplementary Figure 6


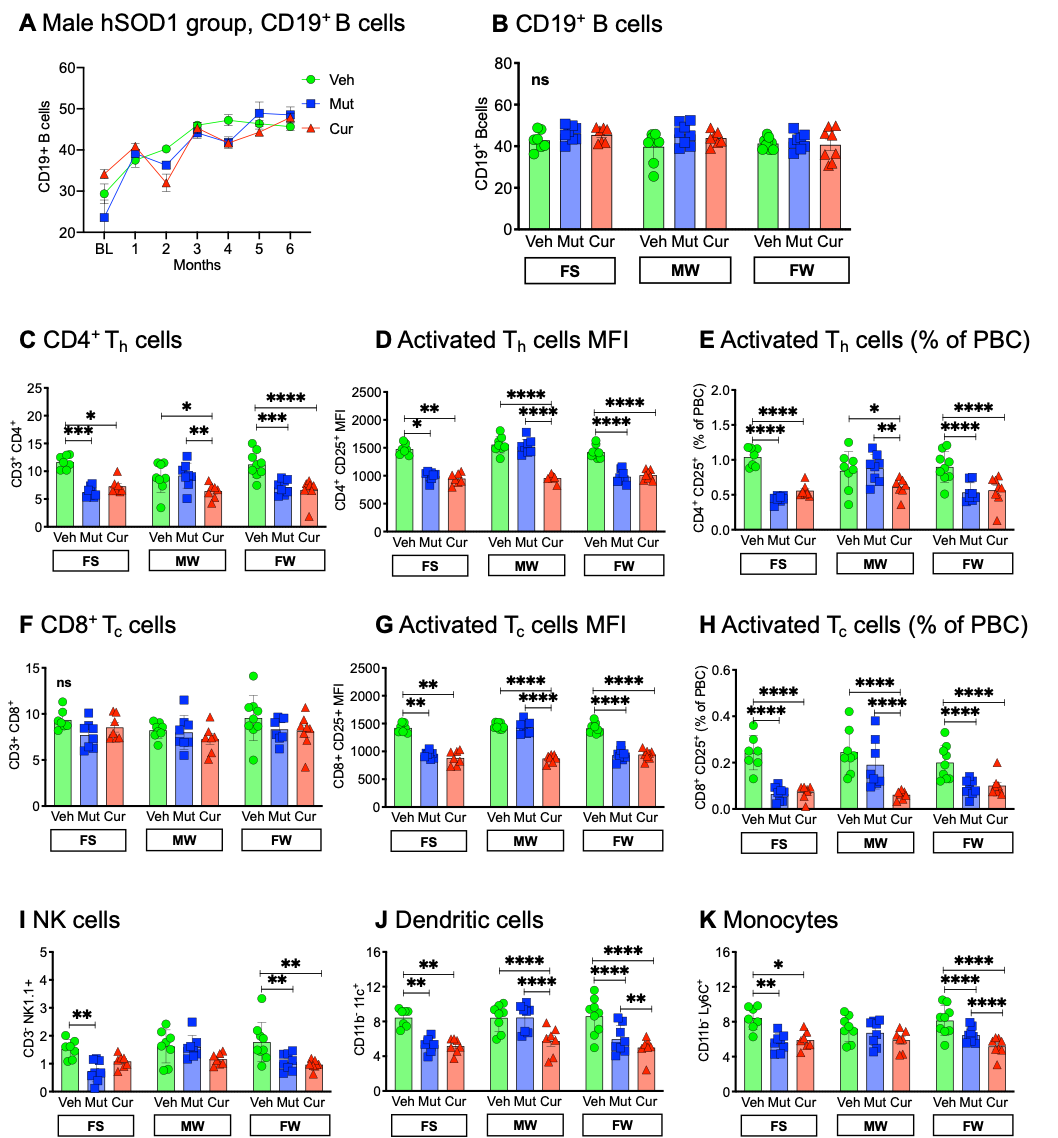


**Supplementary Figure 6: (A)** Within the male hSOD1 cohort, there were no differences in CD19^+^ B cells in peripheral blood between feeding groups for any month, mixed-effects model analyses (REML) with Tukey’s multiple comparisons. **(B-K)** Immunophenotyping of peripheral blood cells for the following groups: female hSOD1 (**FS**), male WT (**MW**) and female WT (**FW**). Mutant and curli-fed females, both FS and FW showed significantly suppressed immune responses **(C,D,E,G,H,I,J,K)**. However, suppressed immune responses in male WT **(C,D,E,G,H,J)** and male hSOD1 (Fig. 7) mice were only observed in response to feeding of curli-producing *E. coli*. *p<0.05, **p<0.01, ****p<0.001, one-way ANOVA. MFI=Geometric Mean Fluorescence Intensity; PBC=Peripheral Blood Cells. n=6-9 per group.

Supplementary Figure 7


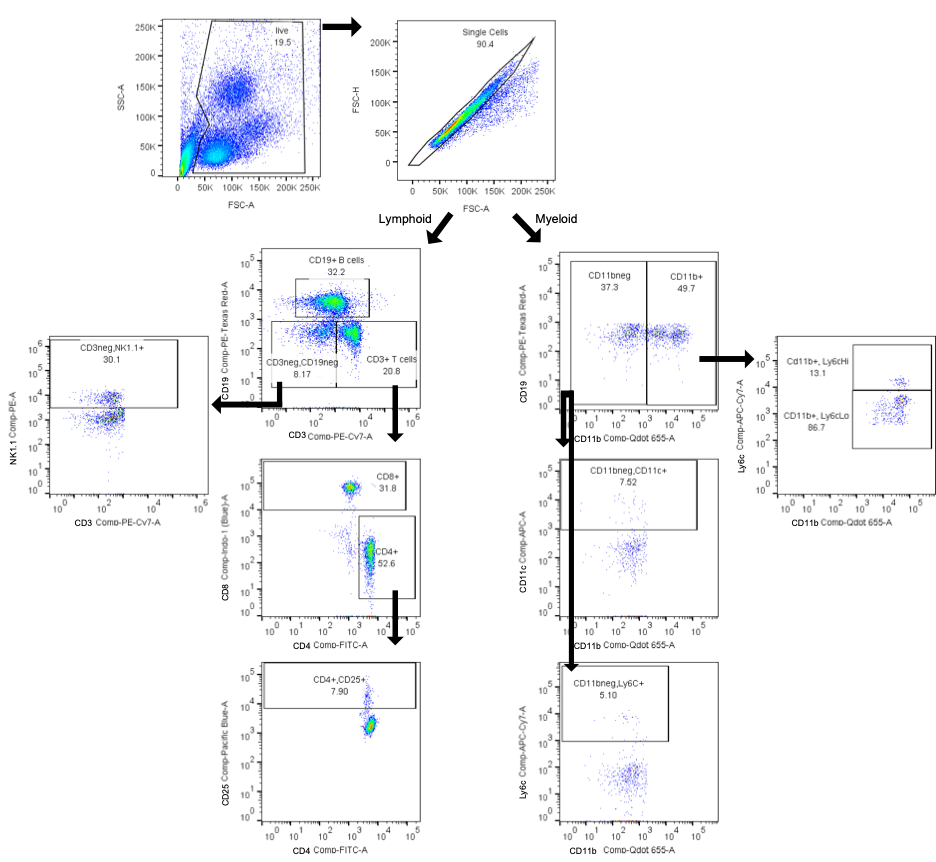


Supplementary Figure 7: Gating strategy for peripheral blood immunophenotyping

Supplementary Figure 8

Supplementary Figure 8: hSOD1-G93A (Full Western Blot Film) for **Figure 4D** and **Supp. Fig. 4D, E, F.**

Supplementary Figure 9

Supplementary Figure 9: TNF𝜶 (Full Western Blot Film) for **Figure 4D** and **Supp. Fig. 4D, E, F.**

Supplementary Figure 10

Supplementary Figure 10: β-actin (Full Western Blot Film) for **Figure 4D** and **Supp. Fig. 4D, E, F.**
